# Adherence to UK Dietary Reference Values and Lower Odds of Non‐Alcoholic Fatty Liver Disease: A Secondary Analysis of a Case–Control Study

**DOI:** 10.1002/edm2.70273

**Published:** 2026-07-01

**Authors:** Mehdi Khakian, Sanaz Jamshidi, Mitra Kazemi Jahromi, Ebrahim Mokhtari, Setareh Alibakhshi, Niloufar Saber, Reza Sadeghi, Ammar Salehi‐sahlabadi, Elham Sobhrakhshankhah, Farshad Teymoori, Parvin Mirmiran, Hossein Farhadnejad

**Affiliations:** ^1^ Nutritional Sciences Research Center Iran University of Medical Sciences Tehran Iran; ^2^ Department of Nutrition, School of Public Health Iran University of Medical Sciences Tehran Iran; ^3^ Center for Cohort Study of Shiraz University of Medical Sciences Employees Shiraz University of Medical Sciences Shiraz Iran; ^4^ Endocrinology and Metabolism Research Center Hormozgan University of Medical Sciences Bandar Abbas Iran; ^5^ Nutrition and Endocrine Research Center, Research Institute for Endocrine Disorders, Research Institute for Endocrine Sciences Shahid Beheshti University of Medical Sciences Tehran Iran; ^6^ Department of Community Nutrition, School of Nutrition and Food Sciences Shiraz University of Medical Sciences Shiraz Iran; ^7^ Faculty of Health Southern Cross University Gold Coast Queensland Australia; ^8^ Clinical Biochemistry Research Center, Basic Health Sciences Institute Shahrekord University of Medical Sciences Shahrekord Iran; ^9^ Department of Community Nutrition, School of Nutrition and Food Science Isfahan University of Medical Sciences Isfahan Iran; ^10^ Gastrointestinal and Liver Diseases Research Center Iran University of Medical Sciences Tehran Iran

**Keywords:** adult, dietary indices, dietary pattern, non‐alcoholic fatty liver disease

## Abstract

**Background/Aim:**

The current study aimed to assess the association between the UK dietary reference values (UK‐DRV) index and odds of non‐alcoholic fatty liver disease (NAFLD).

**Methods:**

This case–control study enrolled 225 newly diagnosed NAFLD patients and 450 controls, aged 20–60 years. Dietary intake was assessed via a validated food frequency questionnaire, and the UK‐DRV index was calculated for all participants. Using multivariable logistic regression, odds ratios (ORs) and 95% confidence intervals (95% CIs) of NAFLD were determined across tertiles of the UK‐DRV index.

**Results:**

The mean ± SD of the UK‐DRV index among control and case groups was 8.84 ± 2.92 and 8.54 ± 2.94, respectively. In the multivariable model, after controlling for potential confounders, the odds of NAFLD were reduced across tertiles of UK‐DRV index (OR: 0.39; 95% CI: 0.20–0.74, P for trend: 0.001). Also, each 1‐SD increase in UK‐DRV index (OR: 0.72; 95% CI: 0.57–0.91, P: 0.007) and its components, including fruits and vegetables (OR: 0.65; 95% CI: 0.50–0.84, P: 0.001) and fibre intake (OR: 0.74; 95% CI: 0.54–0.99, P: 0.048), was inversely associated with odds of NAFLD. Furthermore, each 1‐SD increase in sugar intake as a negative component of the UK‐DRV index was positively associated with odds of NAFLD (OR: 1.52; 95% CI: 1.22–1.90, *p* < 0.001).

**Conclusions:**

Our findings suggest that a diet with a higher score of the UK‐DRV index, characterised by higher intake of fruits, vegetables, and fish and lower intakes of refined carbohydrates, saturated fats, sodium, and simple sugars, may be related to lower odds of NAFLD in adults.

## Graphical abstract

Higher adherence to the UK‐DRV index—reflecting greater intake of fibre, fruits, vegetables, and fish, and lower intake of sugars and saturated fats—is associated with lower odds of NAFLD in Iranian adults, though causality cannot be inferred from this case–control design.

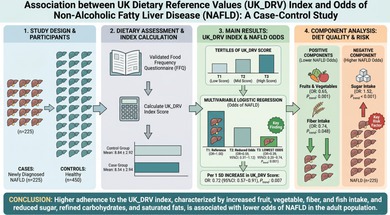

**Abbreviations**
BMI: body mass index
BNF: British Nutrition Foundation
CIs: confidence intervals
COMA: Committee on Medical Aspects of Food Policy
CVDs: cardiovascular diseases
FCT: Food Composition Table
FFQ: food frequency questionnaire
HEI: Healthy Eating Index
HFD: high‐fat/saturated‐fat diets
IPAQ: International Physical Activity Questionnaire
NAFLD: Non‐Alcoholic Fatty Liver Disease
NASH: non‐alcoholic steatohepatitis
NPS: nutrient profiling systems
ORs: odds ratios
PUFAs: polyunsaturated fatty acids
SD: standard deviation
SES: socioeconomic status
SFA: saturated fatty acids
T2D: type 2 diabetes
UK‐DRVs: UK Dietary Reference Values
WC: waist circumference

## 1 Introduction

Non‐Alcoholic Fatty Liver Disease (NAFLD) is a condition characterised by excessive fat accumulation in the liver (steatosis) in individuals who consume little or no alcohol [1]. It ranges from simple fatty liver (steatosis) to more severe forms, including non‐alcoholic steatohepatitis (NASH), fibrosis, cirrhosis, and even liver cancer [1]. NAFLD has become the most common chronic liver disease globally, surpassing alcohol‐related liver disease and viral hepatitis [2]. Its prevalence has risen sharply due to increasing rates of obesity, diabetes, and metabolic syndrome [3]. It is estimated that 25%–30% of the global population has NAFLD (around 1.7–2 billion people) [4]. In Iran, a recent systematic review and meta‐analysis of 31 studies (41,971 participants) estimated the overall prevalence of NAFLD at 33% [5]. Nutrition plays a pivotal role in both the prevention and management of NAFLD [6].

Dietary indices are useful tools for examining the relationship between dietary habits and patterns and the risk of various health outcomes [7]. By incorporating various dietary factors such as nutrients, foods, food groups, and even non‐nutrient components, these indices provide a comprehensive view of the actual diet of individuals, which helps to improve the predictive power of disease risk [8, 9]. Some of these indices have been developed based on dietary guidelines and reference values in different countries and populations. The UK Dietary Reference Values (UK‐DRVs) are guidelines for nutrient intake established by the British Nutrition Foundation (BNF) and the Committee on Medical Aspects of Food Policy (COMA) [10]. They provide recommendations for energy, macronutrients, vitamins, and minerals to maintain good health in different population groups. Based on the UK‐DRV [11], the sixteen‐point UK‐DRV index has been developed and proposed to represent adherence to the Public Health England UK's dietary strategy for optimal health and prevention of cardiovascular diseases (CVDs) [12]. The UK Dietary Policy for the Prevention of CVDs report's six nutritional components, total carbs, sugars, total fat, saturated fatty acid (SFA), salt, and dietary fibre, as well as two food category components, fruit and vegetables combined, and total fish, were used to calculate the UK‐DRV index score [10].

So far, few studies have been conducted on the association of the UK‐DRV index score with the odds of various diseases. In a cross‐sectional study within the framework of the Airwave Health Monitoring study, it was shown that each two‐point increment in the DRV index score is associated with an increase in some metabolic parameters, such as waist circumference (WC), body mass index (BMI), total cholesterol, and HbA1c levels. Furthermore, the risk of type 2 diabetes (T2D) and obesity is directly associated with each one‐point increase in the DRV score [10]. All of these factors can be considered risk factors for fatty liver [1]. Also, a dietary intervention based on the UK Dietary Guidelines, characterised by sodium, total fat, SFA, fruit/vegetables, and whole grains, has been shown to be associated with improvements in BMI, blood pressure, and lipid profile in a UK population [13].

Furthermore, there are other dietary guideline‐based indices and nutrient profiling systems (NPS) that take a similar approach to the UK‐DRV index score, assessing diet quality based on national guidelines and nutrient intake patterns. A nutrient profiling (NP) score, based on UK guidelines, showed an inverse relationship with HbA1c and total cholesterol, but was directly associated with BMI. However, after stratified analysis showed that the association was reversed in both acceptable energy reporters and in those who under‐reported energy intake [10]. Moreover, a well‐known guideline‐based index is the Healthy Eating Index (HEI), which includes various food groups based on the Dietary Guidelines for Americans [14]. Multiple large‐scale studies consistently show that higher HEI scores are associated with a lower odds of NAFLD. Individuals in the highest HEI categories had significantly lower odds of developing these liver diseases compared to those with the lowest scores [15, 16, 17, 18].

According to the current literature, studies on the DRV index are very rare. Also, the efficacy of this index has not been tested in non‐UK populations. Therefore, in the present study, we aimed to investigate the association of this dietary index with the odds of NAFLD in adults using a case–control design.

## 2 Materials and Methods

### 2.1 Study Population

**FIGURE 1:**
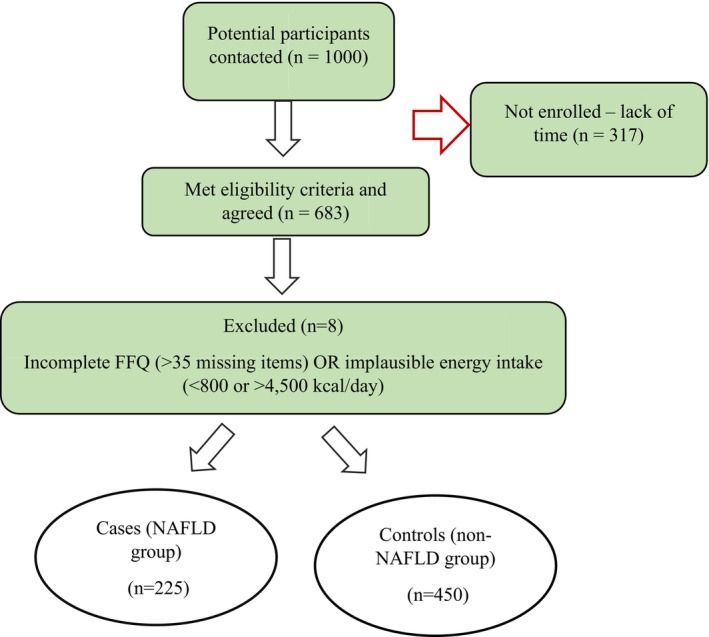
Flowchart showing the participant recruitment and selection process in the case–control study.

This case–control study was carried out at a referral centre for metabolic liver diseases affiliated with Isfahan University of Medical Sciences (the Metabolic Liver Disease Research Center). A total of 675 individuals were enrolled, comprising 225 patients with newly diagnosed non‐alcoholic fatty liver disease (NAFLD) and 450 control subjects. The age range of participants was 20–60 years (Figure 1).

All enrolled individuals underwent abdominal ultrasonography using a GE Logiq S8 system (GE Healthcare, USA) fitted with a convex transducer. A single radiologist with expertise in hepatobiliary imaging performed all examinations and was kept blind to the participants’ dietary intake and other exposure variables relevant to the study hypothesis.

The diagnosis of hepatic steatosis was made based on standard ultrasonographic criteria: Increased echogenicity of the liver relative to the renal cortex, posterior beam attenuation, and poor visualisation of the diaphragm and intrahepatic vessels. Individuals with ultrasonographic evidence of steatosis were categorised as NAFLD cases, provided that other etiologies of liver disease and significant alcohol use were ruled out.

Both cases and controls were clinic‐based and selected via convenience sampling from individuals referred to the same centre for routine abdominal, pelvic, or hepatobiliary ultrasound. The same standardised imaging protocol was applied to all. Controls were required to have no ultrasonographic signs or clinical symptoms suggestive of NAFLD.

Participants were included in the study if they met the following criteria: (1) not following a special diet; (2) no history of cardiovascular disease, kidney or liver disease, diabetes, severe gastrointestinal disorders, malignancy, thyroid disorders, or autoimmune diseases; and (3) no use of hepatotoxic or steatogenic medications. Participants were excluded if they completed fewer than 35 items on the food frequency questionnaire (FFQ) or reported under‐ or over‐reported daily energy intakes (< 800 kcal/day or > 4,500 kcal/day) (eight participants).

### 2.2 Dietary Assessment

Trained dietitians administered a validated 168‐item semi‐quantitative FFQ [19, 20] through face‐to‐face interviews to collect dietary data of the study population during the previous year. As previously described [21], reported portion sizes (in household measures) were converted to grams. Nutrient and energy values were calculated using the USDA Food Composition Table (FCT). For local or indigenous food items that were not available in the USDA FCT, the Iranian FCT was used.

### 2.3 Dietary Reference Values Index Scoring

The DRV index score was calculated using the consumption levels of six nutritional components—total fat, saturated fatty acids, total carbohydrates, sugars, salt, and dietary fibre—as well as two food group components: Combined fruits and vegetables intake, and total fish intake [10]. These components were selected based on the recommendations outlined in the UK Dietary Policy for the Prevention of Cardiovascular Disease (CVD) report. The DRV index scoring system was constructed following a previously established approach [10].

Each component was assigned 0, 1, or 2 points according to predefined intake cutoffs based on UK dietary recommendations, and the component scores were summed to generate a total score from 0 to 16, with higher scores reflecting greater adherence to the UK dietary guidelines. This composite score was derived from all consumed foods and beverages, excluding alcohol.

### 2.4 Assessment of Other Variables

A standardised demographic questionnaire was used to collect socio‐demographic data, including age, sex, smoking status, educational attainment, marital status, and socioeconomic status (SES). Three variables were used to calculate the SES score [22]: Family size (≤ 4 or > 4 members), education level (college‐educated vs. non‐college‐educated), and home ownership status (owner vs. non‐owner). For each variable, individuals were assigned a score of 1 if they met the following criteria: (1) family size of 4 or fewer members; (2) possession of a college degree; or (3) ownership of a home. The overall SES score was obtained by summing these individual scores. Based on the total, participants were categorised as having low SES (score of 0 or 1), moderate SES (score of 2), or high SES (score of 3). In the present study, smoking status was categorised as either ‘yes’ or ‘no’. The ‘yes’ group included current daily smokers, occasional smokers, and former smokers. The ‘no’ group consisted of never smokers.

The International Physical Activity Questionnaire (IPAQ) was administered through face‐to‐face interviews to assess participants' physical activity levels [23]. All results were expressed in metabolic equivalents per week (METs/week) [24, 25]. Participants' body weight was measured using digital scales (Seca, Hamburg, Germany) with an accuracy of 100 g, with participants wearing light clothing and no shoes. Height was measured in a standing position without shoes using a stadiometer with a precision of 0.5 cm. Body mass index was then calculated by dividing weight in kilograms by the square of height in meters (kg/m^2^).

### 2.5 Statistical Analysis

All analyses were performed using SPSS version 15.0 (SPSS Inc., Chicago, IL, USA). Data normality was assessed through both visual inspection (histograms) and the Kolmogorov–Smirnov test. Continuous variables are presented as mean ± standard deviation (SD) for normally distributed and median with interquartile range (25th–75th percentiles) for non‐normal distributions. Also, categorical variables are expressed as percentages. Participants were categorised into tertiles based on the UK_DRV index. Trends across tertiles of the UK‐DRV index were analysed using linear regression, while trends for categorical variables were determined with the chi‐square test. Multivariable logistic regression was performed to estimate the odds ratios (ORs) and 95% confidence intervals (CIs) for NAFLD across tertiles of the UK‐DRV index. Four models were used: Crude model, Model 1 (adjusted for age and sex), Model 2 (further adjusted for smoking status, physical activity, SES, marital status, and energy intake), and Model 3 (further adjusted for BMI). Also, odds ratio (95% CI) of NAFLD for each SD increment of UK‐DRV index and its components, including carbohydrate (% of energy), fibre (g/d), fruits & vegetables (g/d), fish (g/d), sodium (mg/d), fat (% of energy), saturated fat (% of energy), sugar (% of energy) were determined. Multicollinearity among the covariates included in the regression models was evaluated by calculating the variance inflation factor (VIF) and tolerance values; no problematic multicollinearity was detected. A *p*‐value of less than 0.05 was considered statistically significant.

## 3 Results

The mean ± SD age and BMI in the participants of the control group were 37.9 ± 8.9 years and 25.0 ± 3.09 kg/m^2^, respectively. Also, the mean ± SD age and BMI in the case group were 38.6 ± 8.7 years and 30.5 ± 2.94 kg/m^2^, respectively. The mean ± SD of the UK‐DRV index in individuals of the control and case groups was 8.84 ± 2.92 and 8.54 ± 2.94, respectively.

The study population characteristics across the tertiles of the UK‐DRV index were reported in Table 1. The mean age and energy intake of individuals in the third tertile of the UK‐DRV index were higher than those in the first tertile (*p* < 0.05). However, no differences were observed in the percentage distribution of gender, percentage of smokers, marital status, SES, or the mean BMI and physical activity according to the tertiles of the UK‐DRV index. Table 1 also showed that the intake of fibre, fish, fruits, and vegetables—as positive components of the UK‐DRV index—was higher in individuals in the highest tertile of the UK‐DRV index compared to those in the lowest tertile, whereas the intake of carbohydrates, sodium, total fat, saturated fat, and simple sugars—as negative components of the UK‐DRV index—was lower in individuals in the highest tertile compared to the lowest tertile of this dietary index.

**TABLE 1:** Population characteristics based on the tertiles of the UK Dietary Reference Values Index.

Variables	Tertiles of the UK Dietary Reference Values Index			P‐trend
	T_1_ (*n* = 291)	T_2_ (*n* = 237)	T_3_ (*n* = 147)	
Demographic data				
Age, (year)	36.8 ± 8.45	38.0 ± 8.74	40.8 ± 9.24	< 0.001
Sex (male)	48.5	58.6	53.1	0.065
Body mass index (Kg/m^2^)	26.6 ± 4.03	26.9 ± 4.50	27.1 ± 4.55	0.228
Physical activity (MET/h/week)	1,465 ± 853	1,448 ± 908	1,345 ± 890	0.262
Smoking (yes)	3.8	5.1	3.4	0.674
Marital status (married, %)	83.8	84.0	83.7	0.997
Socio‐economic status				0.286
Low	29.9	27.0	26.5	
Middle	37.5	40.9	35.4	
High	32.6	32.1	38.1	
Energy intake (Kcal/d)	2,154 ± 607	2,242 ± 644	2,308 ± 606	0.011
UK‐DRV index components				
Carbohydrate (% of energy)	52.7 ± 6.57	59.2 ± 5.00	63.4 ± 5.03	< 0.001
Fibre (g/d)	16.2 ± 6.55	20.8 ± 7.66	25.0 ± 7.38	< 0.001
Fruits & Vegetables (g/d)	484.4 ± 237.2	673.8 ± 302.9	897.5 ± 387.2	< 0.001
Fish (g/d)	6.37 (3.42–11.80)	6.74 (3.53–13.51)	7.50 (4.33–15.0)	0.001
Sodium (mg/d)	3,538 (2,731–5,753)	3,523 (2,482–5,753)	3,184 (2,242–4,587)	0.026
Fat (% of energy)	36.0 ± 6.73	29.5 ± 4.94	26.0 ± 4.10	< 0.001
Saturated fat (% of energy)	12.5 ± 3.06	9.77 ± 2.13	8.39 ± 1.55	< 0.001
Sugar (% of energy)	10.7 ± 6.35	8.56 ± 5.33	6.89 ± 3.36	< 0.001
UK DRV	5.91 ± 1.89	10.0 ± 0.76	12.2 ± v 0.62	< 0.001

*Note:* Data were expressed as mean (SD) or median (interquartile range) and per cent (%) for continuous and categorical variables, respectively.

Abbreviation: UK DRV, UK Dietary Reference Values.

The OR (95% CIs) of NAFLD across tertiles of the UK‐DRV index are reported in Table 2. In model 1, adjusted for age and sex, no significant association was observed between the UK‐DRV index and odds of NAFLD (OR: 0.72; 95% CI: 0.47–1.20, P for trend: 0.089). However, in the final multivariable model, after adjustment for age, sex, BMI, smoking, physical activity, marital status, SES, and dietary intake of energy, the odds of NAFLD were decreased across tertiles of UK‐DRV index (OR: 0.39; 95% CI: 0.20–0.74, P for trend: 0.001).

**TABLE 2:** Odds ratio (95% CI) of non‐alcoholic fatty liver disease across tertiles of the UK Dietary Reference Values index.

Dietary indices	OR (95% CI) of NAFLD			
	Tertile 1	Tertile 2	Tertile 3	P trend^a^
UK‐DRV				
Median score	6.0	10.0	12.0	
Case/Controls	106/185	74/163	45/102	
Crude model	1.00 (Ref)	0.79 (0.55–1.14)	0.77 (0.50–1.17)	0.146
Model 1^a^	1.00 (Ref)	0.76 (0.53–1.10)	0.72 (0.47–1.20)	0.089
Model 2^b^	1.00 (Ref)	0.67 (0.45–1.00)	0.58 (0.36–0.92)	0.011
Model 3^c^	1.00 (Ref)	0.50 (0.29–0.84)	0.39 (0.20–0.74)	0.001

Abbreviations: SD, standard deviation; UK DRV, UK Dietary Reference Values.

^a^ Model 1: Adjusted for age and sex.

^b^ Model 2: Additionally adjusted for model 1 and smoking, physical activity, marital status, socio‐economic status, and dietary intake of energy.

^c^ Model 3: Additionally adjusted for model 2 and body mass index.

Odds ratio (95% CI) of NAFLD for each standard deviation increment of the UK‐DRV index and its components was indicated in Table 3. Our results showed that each one SD increase in UK‐DRV index (OR: 0.72; 95% CI: 0.57–0.91, *p*‐value: 0.007) and its components including fruits & vegetables (OR: 0.65; 95% CI: 0.50–0.84, *p*‐value: 0.001) and fibre intake (OR: 0.74; 95% CI: 0.54–0.99, *p*‐value: 0.048) was associated with lower odds of developing NAFLD. However, each one SD increase in sugar intake (% of energy) as a negative component of the UK‐DRV index was associated with higher odds of NAFLD (OR: 1.52; 95% CI: 1.22–1.90, *p*‐value < 0.001). The association of other components of the UK‐DRV index with odds of NAFLD was not statistically significant.

**TABLE 3:** Odds ratio (95% CI) of NAFLD for each standard deviation increment of the UK Dietary Reference Values index and its components.

UK‐DRV components	OR (95% CI) of NAFLD	*p*
CarBohydrate (% of energy), SD = 7.2		
Crude model	0.98 (0.84–1.15)	0.870
Adjusted Model*	0.94 (0.75–1.19)	0.650
Fibre (g/d), SD = 7.9		
Crude model	1.13 (0.96–1.32)	0.122
Adjusted Model*	0.74 (0.54–0.99)	0.048
Fruits & Vegetables (g/d), SD = 328		
Crude model	0.89 (0.76–1.05)	0.186
Adjusted Model*	0.65 (0.50–0.84)	0.001
Fish (g/d), SD = 8.6		
Crude model	0.98 (0.84–1.16)	0.897
Adjusted Model*	0.82 (0.64–1.06)	0.137
Sodium (mg/d), SD = 2616		
Crude model	0.99 (0.85–1.17)	0.985
Adjusted Model*	0.90 (0.70–1.15)	0.378
Fat (% of energy), SD = 6.9		
Crude model	0.98 (0.83–1.15)	0.822
Adjusted Model*	1.05 (0.84–1.32)	0.650
Saturated fat (% of energy), SD = 3.0		
Crude model	0.90 (0.76–1.06)	0.216
Adjusted Model*	0.99 (0.96–1.01)	0.913
Sugar (% of energy), SD = 5.6		
Crude model	1.55 (1.31–1.83)	< 0.001
Adjusted Model*	1.52 (1.22–1.90)	< 0.001
UK‐DRV, SD = 2.9		
Crude model	0.90 (0.77–1.05)	0.211
Adjusted Model*	0.72 (0.57–0.91)	0.007

Abbreviations: SD, standard deviation; UK DRV, UK Dietary Reference Values.

* Adjusted Model: Adjusted for age, sex, body mass index, smoking, physical activity, marital status, socio‐economic status, and dietary intake of energy.

## 4 Discussion

In the current study, we sought to explore the relationship between the UK‐DRV index score and the probability of developing NAFLD. Our results indicated that after adjusting for potential confounding variables, the odds of NAFLD were found to decrease across the UK‐DRV tertiles. Overall, the intake of fibre, fruits, and vegetables was associated with a lower risk, while a higher intake of sugars was correlated with a higher odds of NAFLD.

Since diet consists of various food and nutrient exposures, analysing dietary patterns, done based on the nutritional index score (such as the DRV index score), is viewed as an alternative method to explore the relationship between diet and disease risk in epidemiological research. This method looks at the overall diet and takes into account the combined effects of food and nutrient intake, which may provide a better prediction of disease risk than focusing on single foods or nutrients [26]. In adults, diet quality has been utilised to measure the risk of chronic diseases, including CVDs, certain types of cancer, and both overall and disease‐specific mortality. Diet quality indices, or scores, such as the UK‐DRV index score, serve as instruments that offer a comprehensive rating on a numerical scale, reflecting an individual's dietary consumption in relation to nutrient and/or dietary recommendations [27]. In this regard, a previous study showed that the DRV index score correlates with the overall quality of diet and the risk factors for CVDs and T2D, thereby endorsing its use in nutritional epidemiological research focused on CVD risk [10].

To investigate the impact of each component of the UK‐DRV index score, our data show an inverse relationship between fibre intake and NAFLD risk, echoing robust nutritional evidence. A U.S. cross‐sectional analysis found that individuals in the highest fibre tertile had lower odds of NAFLD. In this study, higher cereal, fruit, and vegetable fibre intakes were associated with substantial reductions in odds of NAFLD [28]. Another nationwide cross‐sectional investigation revealed that compared to those in the lowest tertile, participants in the highest tertile of dietary fibre intake had a reduced likelihood of developing NAFLD (OR = 0.81). In addition, mediation analysis revealed that the protective effect of dietary fibre on NAFLD was fully mediated by obesity. Also, higher fibre intake was associated with improvements in liver function indicators. These results suggest that increasing dietary fibre consumption may offer substantial protection against NAFLD [29]. Mechanistically, fibre may benefit hepatic health via improved insulin sensitivity, enhanced lipid excretion, and modulation of gut microbiota and short‐chain fatty acids [30, 31] https://www.frontiersin.org/journals/nutrition/articles/10.3389/fnut.2020.593735/full?utm_source=chatgpt.com. Higher intakes of fruits and vegetables—observed in the highest UK‐DRV index score tertile—were also associated with lower odds of NAFLD, consistent with meta‐analyses of observational studies. A recent study of 11 such studies involving nearly 500,000 adults found that high vegetable intake was associated with a 22% lower NAFLD risk (OR 0.78), while high fruit intake led to a ~12% risk reduction (OR 0.88) [30]. The inverse association of fruits and vegetables is likely due to their fibre, antioxidants (e.g., vitamins C, polyphenols), and anti‐inflammatory phytochemicals, which together improve hepatic metabolism and lower oxidative stress and inflammation [32, 33]. Thus, our finding that higher fibre, fruit and vegetable consumption—captured by the UK‐DRV index score—lowers NAFLD odds is well‐supported.

In the current study, fish intake increased across tertiles of UK‐DRV index scores in participants. As a key component of the UK‐DRV index, higher fish intake contributes to the diet's overall association with a lower odds of NAFLD in individuals with a higher score of the UK‐DRV index. Fish, especially oily varieties rich in omega‐3 polyunsaturated fatty acids (PUFAs), have well‐documented hepatoprotective properties. Omega‐3 PUFAs improve hepatic lipid metabolism by reducing de novo lipogenesis, enhancing fatty acid oxidation, and modulating inflammatory pathways [34, 35]. Furthermore, fish consumption may beneficially influence gut microbiota composition and reduce endotoxemia, both of which are implicated in NAFLD pathogenesis [36, 37]. Our results emphasise that promoting fish intake within dietary guidelines such as the UK‐DRV could be a practical strategy to mitigate NAFLD risk.

Conversely, our study observed a decrease in sodium consumption in participants within the highest UK‐DRV index score tertile, which aligns with growing evidence implicating excessive sodium intake in the pathogenesis of NAFLD. A population‐based study in the US revealed that elevated sodium intake was linked with higher odds of NAFLD [38]. Mechanistically, excessive sodium intake can promote oxidative stress and inflammatory pathways through activation of the renin‐angiotensin‐aldosterone system, potentially accelerating hepatic dysfunction [39]. Moreover, dietary sodium often serves as a proxy for consumption of ultra‐processed foods, which are high in sodium, sugars, and unhealthy fats, all contributing to NAFLD risk [40]. It should be noted that diets high in sodium have been demonstrated to raise leptin levels and insulin resistance, a key marker of body fatness [41]. Also, participants in the highest UK‐DRV index score tertile consumed lower amounts of simple sugars and saturated fats, aligning with findings linking high sugar intake; a meta‐analysis has shown a significant positive correlation between increased intake of sugar‐sweetened beverages (SSB) and NAFLD in both genders [42]. It is believed that sugar‐sweetened beverages increase hepatic de novo lipogenesis, inflammation, and NAFLD progression [43]. In addition, dietary fat could be a significant modifiable element in the progression of NAFLD and NASH [44]. Various studies have shown that high‐fat/saturated‐fat diets (HFD) elevate liver fat levels and contribute to hepatocyte damage [45, 46].

Our study has several notable strengths. First, we introduced a novel index‐based dietary assessment tool, the UK‐DRV index score, which moves beyond the analysis of isolated nutrients to holistically evaluate overall dietary quality and adherence to national dietary recommendations. This approach more accurately reflects the complex, synergistic ways in which foods are consumed in a real‐world setting. Nutritional intake and physical activity data were assessed using questionnaires that have been previously validated for use in the Iranian population. Furthermore, the robustness of our findings is enhanced by the careful adjustment for a wide array of potential confounders, including key demographic, clinical, and lifestyle factors, which increase the internal validity of the observed associations.

Several limitations of this study should be acknowledged. First, the case–control design precludes any conclusion about causality. A common concern in such designs is reverse causation—i.e., the disease itself affecting dietary recall or habits. However, because we restricted the case group to individuals with newly diagnosed (incident) NAFLD, the likelihood of reverse causation (dietary modification due to pre‐existing illness) is considerably reduced. Second, selection bias may be present, since controls were drawn from the same clinical population as cases. Third, the use of a food frequency questionnaire (FFQ) for dietary assessment is inherently susceptible to recall bias and measurement error, as it depends on participants' long‐term memory. Although this limitation cannot be completely eliminated, we used a validated FFQ to mitigate its impact. Fourth, despite careful adjustment for a wide range of confounders, residual confounding from unknown or unmeasured variables (e.g., supplement use, sleep quality, or genetic predisposition) cannot be entirely ruled out. Fifth, misclassification of the outcome represents another potential limitation. Although ultrasound is a standard tool for diagnosing NAFLD, its sensitivity is limited, especially for mild hepatic steatosis. As a result, some participants with mild steatosis might have been incorrectly classified as controls (non‐differential misclassification). This type of error typically biases effect estimates toward the null. Sixth, the findings may have limited generalisability. This investigation was performed at a single referral centre in Iran, so the sample may not be fully representative of the wider population. Referral and selection biases are possible because individuals attending this centre might differ from those in other medical settings or the general community. On the other hand, conducting the study at a referral centre enhances internal validity by ensuring standardised diagnostic protocols and minimising heterogeneity. Moreover, we excluded participants with certain concurrent conditions (e.g., viral hepatitis, alcoholic liver disease, other chronic liver disorders) to reduce confounding. Although this exclusion improves internal validity, it may restrict the applicability of our results to NAFLD patients who have these comorbidities. Consequently, caution is needed when extrapolating our findings to other populations or clinical settings. The analyses of individual UK‐DRV components were exploratory and should be interpreted cautiously because they may be affected by type I error due to multiple comparisons.

## 5 Conclusions

Our findings suggest that a diet rich in fibre, fruits, vegetables, and fish, but low in sugars, saturated fats, and sodium, may be associated with reduced odds of NAFLD. The UK‐DRV index appears useful for quantifying this protective pattern. However, due to the observational design, causality cannot be inferred. The index may still aid clinical risk assessment and public health strategies, pending confirmation in prospective studies.

## Author Contributions

**Mehdi Khakian:** conceptualization, formal analysis, writing – review and editing, writing – original draft, methodology. **Setareh Alibakhshi:** formal analysis, writing – review and editing. **Reza Sadeghi:** writing – original draft, writing – review and editing. **Elham Sobhrakhshankhah:** writing – original draft. **Farshad Teymoori:** supervision, conceptualization, methodology, formal analysis. **Niloufar Saber:** writing – original draft, writing – review and editing. **Ebrahim Mokhtari:** writing – original draft, writing – review and editing. **Sanaz Jamshidi:** writing – original draft. **Ammar Salehi‐sahlabadi:** formal analysis, methodology. **Hossein Farhadnejad:** supervision, conceptualization, formal analysis, writing – original draft, writing – review and editing, methodology. **Parvin Mirmiran:** conceptualization, methodology, writing – review and editing. **Mitra Kazemi Jahromi:** conceptualization, methodology.

## Funding

The authors have nothing to report.

## Ethics Statement

All procedures performed in studies involving human participants adhered to the ethical standards of the institutional and/or national research committee, and to the 1964 Helsinki Declaration and its later amendments or comparable ethical standards. The study was approved by the Ethics Committee of Isfahan University of Medical Sciences, Isfahan, Iran.

## Consent

Informed written consent was obtained from participants.

## Conflicts of Interest

The authors declare no conflicts of interest.

## Data Availability

The data that support the findings of this study are available from the corresponding author upon reasonable request.
